# Detecting population stratification using related individuals

**DOI:** 10.1186/1753-6561-3-s7-s106

**Published:** 2009-12-15

**Authors:** Anthony L Hinrichs, Robert Culverhouse, Carol H Jin, Brian K Suarez

**Affiliations:** 1 Department of Psychiatry, Washington University School of Medicine, 660 South Euclid, Campus Box 8134, St. Louis, Missouri 63110 USA; 2 Department of Medicine, Washington University School of Medicine, 660 South Euclid, St. Louis, Missouri 63110 USA; 3 Department of Genetics, Washington University School of Medicine, 660 South Euclid, St. Louis, Missouri 63110 USA

## Abstract

Although identification of cryptic population stratification is necessary for case/control association analyses, it is also vital for linkage analyses and family-based association tests when founder genotypes are missing. However, including related individuals in an analysis such as EIGENSTRAT can result in bias; using only founders or one individual per pedigree results in loss of data and inaccurate estimates of stratification. We examine a generalization of principal-component analyses to allow for the inclusion of related individuals by down-weighting the significance of individual comparisons.

## Background

At the heart of all genetic case/control association analyses lies estimation of allele frequencies. For linkage analyses and pedigree-based association analyses, allele frequency estimates are used when a parental genotype is missing. Because cryptic population stratification results in misestimates of allele frequency, this can lead to false positives for any type of analysis with missing founder genotypes [1,2]. Current methods for identifying and controlling population stratification rely on unrelated individuals. When they are applied to pedigree data, only the founders are analyzed. This suggests that the situation in which detection of population stratification is most needed is the least tractable with current methods.

Several methods for detecting population stratification exist. Two of the most common methods are implemented in STRUCTURE and EIGENSTRAT [3,4]. The program STRUCTURE uses a Markov-chain Monte Carlo (MCMC) method to identify natural population clusters based on multilocus genotypes. It provides probability of membership for each sample that provides a very natural interpretation. However, it is too computationally intensive to be used on genome-wide association study (GWAS) data involving hundreds of thousands or millions of markers [4]. To handle this volume of data, a more computationally simple method is required. The program EIGENSTRAT uses a very fast linear algorithm to identify population structure. In particular, it performs a principal-component analysis (PCA) on a matrix X; an *M *× *N *matrix (where *M *is the number of markers and *N *is the number of individuals). An eigenvalue decomposition is then performed on the *N *× *N *correlation matrix and population membership is inferred from the eigenvectors. One determines how many natural ethnicities are present by examining the sizes of the eigenvalues using a graphical scree analysis or a numeric approach (such as the recently developed "acceleration factor" [5]).

However, the nature of the eigenvalue decomposition introduces problems when individuals are related. Because biologically related individuals are already genetically correlated, this can bias the decomposition, especially in the presence of a large number of related individuals (such as in large pedigrees). Using only unrelated individuals limits the analysis to either the founders or a sampling of unrelated individuals. Although the founders provide all of the genetic variation present in the subsequent generations and therefore represent all available information, using randomly sampled unrelated individuals results in a loss of information.

## Methods

To allow for the analysis of related individuals, we will apply a recently developed method of linear dimensionality reduction [6]. This method can be considered a generalization of PCA, or "weighted" PCA. In particular, consider a re-formulation of PCA as linear projection from a higher dimensional to a lower dimension space in which we maximize the sum of projected pairwise squared distances:

If we instead consider a system of weights *w*, we can instead maximize

providing a weighted version of PCA. In particular, we define the Laplacian to be an *N *× *N *matrix such that

One then performs an eigenvalue decomposition on the matrix

The use of the Laplacian causes an important change in the process. The PCA is normally computed on the matrix

an *N *× *N *symmetric matrix of the pairwise genetic covariance between subjects. However, since the Laplacian is an *N *× *N *matrix placed within the covariance calculation, we then produce an *M *× *M *symmetric matrix of the weighted pairwise covariance between markers. With dense SNP genotyping, we typically see more markers than individuals by several orders of magnitude, and this computation would be nearly intractable. However, since the Laplacian matrix is positive semidefinite, we can compute a Cholesky decomposition such that

And thus,

Let

We then see that

But then we can compute the eigenvalue decomposition of

This is much more manageable. Further, the eigenvalues for these two are the same and the eigenvectors of the original formulation are simply the product of *Y*^*T *^and the second set of eigenvectors (followed by normalization) [7].

For our analyses, we use a weight based on work by McPeek and colleagues [8]. In particular, they demonstrate the use of the kinship matrix to derive the best linear unbiased estimate (BLUE) of allele frequencies in samples of related individuals.

For *N *individuals, let *K *be the *N *× *N *kinship matrix and let **1 **denote a column vector of length *N *of 1 values. Then the vector

provides the best linear weights to compute allele frequencies for related individuals. In a fully typed pedigree, each founder is given a weight "1" and all other individuals are given weight "0." In any pedigree with a single typed individual, that individual is given weight "1." In the simple case of a nuclear pedigree with *S *children without genotyped parents, each child is given weight 2/(*S*+1). Note that as the number of typed children increase, the sum of the weights tends toward 2 - precisely the number of founders of the pedigree. This generalizes to any sized pedigree; namely, the total of the weights cannot be larger than the number of founders, since the founders were the only source of genetic material in the pedigree.

For pairwise weights between two individuals, we use the product of the individual weights. In particular, we derive *L *from the weight matrix

We test this method compared with the standard EIGENSTRAT method using the Framingham Heart Study data. After cleaning, the Framingham Heart Study data consists of 1180 pedigrees, including 418 singletons. The remaining 762 pedigrees have an average of 8.3 genotyped individuals, including 9 pedigrees with more than 50 genotyped individuals. The best standard of comparison would be an analysis using all founder genotypes, but because not all founder genotypes are available, we apply an algorithm to identify the maximal set of unrelated individuals. We consider the resulting population membership as the "gold standard." We also consider the set of singletons and one individual chosen at random from each pedigree. Finally, we consider the full sample with all related individuals using the standard EIGENSTRAT method and our novel method. We then assess to what degree including related individuals influences the standard method and how well the novel method reproduces the "gold standard." We also examine the total weight of all the genotypes as a measure of how much information is used.

## Results

We used the full 50 k marker set but kept only autosomal SNPs with a minor allele frequency greater than 0.05 and a genotyping rate greater than 99%, for a total of 31,068 SNPs. We dropped individuals with more than 5% missing genotypes, for a total of 6757 individuals.

We considered five data sets for PCA: MaxUnrel, maximum number of unrelateds, our gold standard; Singletons, individuals without genotyped relatives; One per, one individual per pedigree chosen at random; Full, all individuals without weighting; and Weighted, all individuals with weighted PCA as described above. Table 1 reports number of individuals and scaled values of the first three principal components (scaled so PC1 = 1.0). Note that for the two smallest samples, there is evidence of two separate axes of stratification, but this disappears for the larger samples.

**Table 1:** Number of effective individuals for five samples and scaled principal components

Data set^a^	Individuals	PC1	PC2	PC3
MaxUnrel	2014	**1.0000^b^**	0.5113	0.405
Singletons	418	**1.0000**	**0.7045**	0.4808
OnePer	1180	**1.0000**	**0.7074**	0.4475
Full	6757	**1.0000**	0.4002	0.3717
Weighted	2898.7	**1.0000**	0.4147	0.3652

^a^MaxUnrel, maximum number of unrelateds; Singletons, individuals without genotyped relatives; One per, one individual per pedigree chosen at random; Full, all individuals without weighting; and Weighted, all individuals with weighted PCA

^b^Bold components were found significant by the acceleration method.

**Figure 1:**
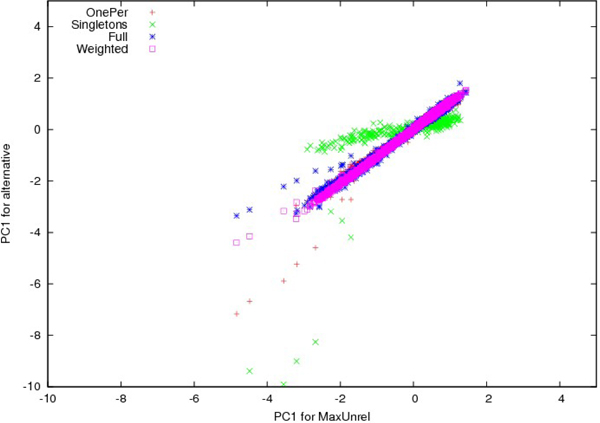
**PC1 for multiple samples**. Consistency of normalized PC1 for subsets compared with maximal set of unrelateds.

Figure 1 shows a plot of the first PC for MaxUnrel compared to the other four samples after scaling to mean zero and SD 1. Only individuals used directly are plotted. Note that the smallest sample (Singletons) shows a clear bias compared with MaxUnrel. We also see handfuls of outliers for all samples, but the weighted method stays closest to MaxUnrel.

**Figure 2:**
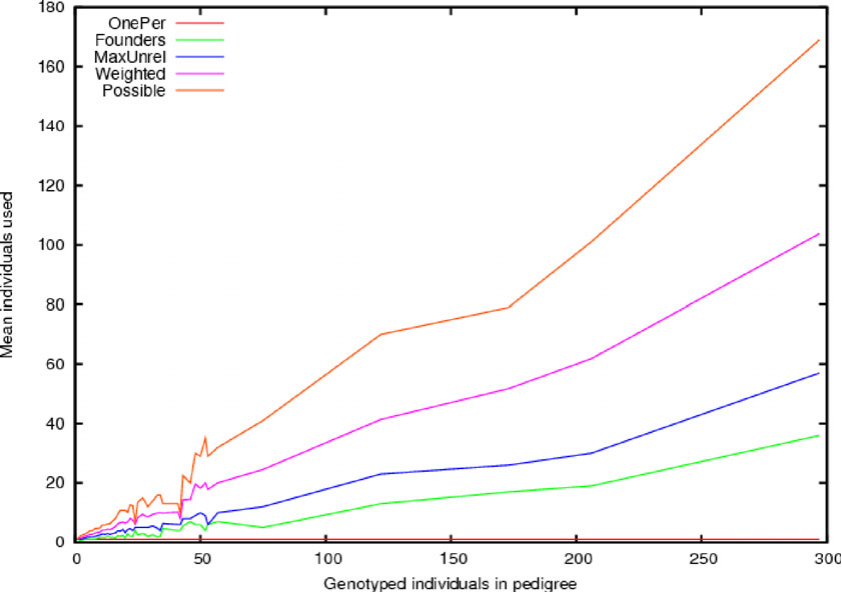
**Samples used**. Mean number of individuals used for number of typed individuals. Possible indicates the mean number of founders (typed or untyped).

Figure 2 shows the mean number of individuals used for each analysis compared with number of genotyped individuals. The novel weighting method shows a clear advantage, especially when pedigrees are very large. This is still substantially less than the "Possible" (the total number of founders, typed or untyped), but much better than using only the typed founders or finding a maximal set of unrelated individuals.

## Discussion

We propose the use of weighted PCA implemented through the presence of a Laplacian matrix to allow detection of stratification in related individuals. Our results indicate the methodology developed by McPeek and colleagues to compute allele frequencies in related individuals can be extended to detection of ethnic stratification. This method uses all available genotypic data, with an effective sample size that approaches the number of founders in the pedigrees. This exceeds other methods of selecting unrelated individuals. Furthermore, we see evidence of bias and outliers when using small subsets of individuals. Using too few individuals for stratification may also artificially inflate evidence of stratification. It does appear that the presence of related individuals in a very large sample seems to have little effect on the stratification analysis, but this might not hold in other circumstances. Furthermore, this method has only been tested on a European American sample with a single principal component (probably identifying a continuous population spread such as northern to southern European). Because the Framingham data does not have any obvious discrete clusters, this method still must be tested in a more diverse population.

## List of abbreviations used

GWAS: Genome-wide association study; MCMC: Markov-chain Monte Carlo; PCA: Principal component analysis.

## Competing interests

The authors declare that they have no competing interests.

## Authors' contributions

ALH developed the method and performed analyses. RC and BKS assisted in design and methodology. CHJ assisted in statistics and data management. All authors read and approved the final manuscript.
